# Modeling the production of belly button lint

**DOI:** 10.1038/s41598-018-32765-9

**Published:** 2018-09-27

**Authors:** P. Deepu

**Affiliations:** Department of Mechanical Engineering, Indian Institute of Technology Patna, Bihta, 801103 Bihar India

## Abstract

We show that respiratory cycle provides a periodic traction force for the production of belly button lint or navel fluff. The relative motion induced between clothing and skin, during breathing, transports the clothing fibers over the abdominal skin via an asymmetric sliding mechanism effected by the specific orientation of the cuticle scales of body hair. The source of these fibers can be the piece of clothing worn adjacent the navel area or the drying towel used after shower. The ratchet like topology of hair surface ensures a net unidirectional transport of these fibers. Since the predominant direction of growth of hair in the abdomen is toward the navel, this unidirectional transport leads to a perpetual accumulation of fibers in the navel over the course of the day. By analyzing the force balance on a moving fiber and the transport dynamics of its number density distribution, we develop a mathematical model to describe the accretion rate of lint fibers in the navel.

## Introduction

**Figure 1:**
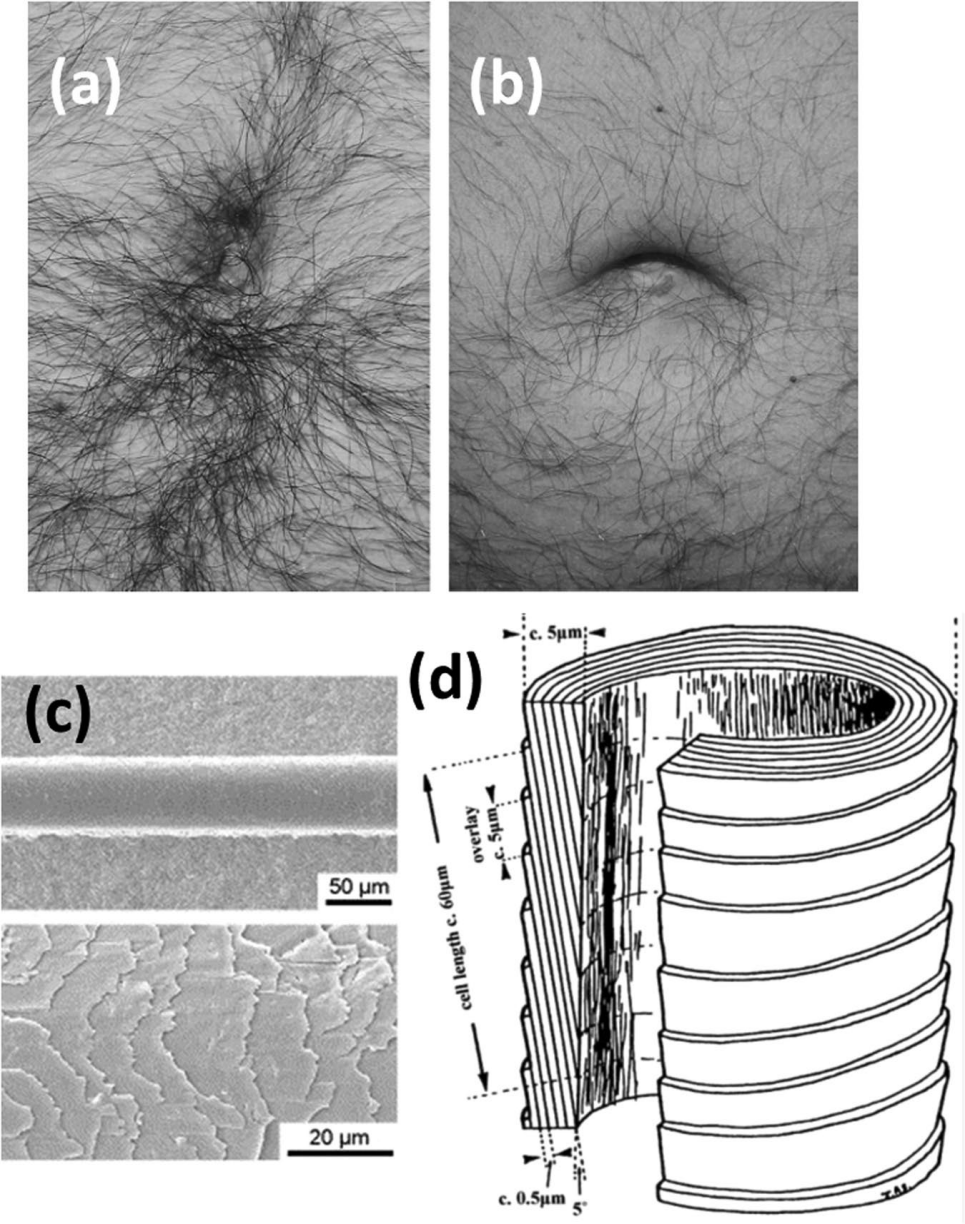
(**a**) A typical navel that produces BBL. **(b**) A typical navel that does not produce BBL. Note the difference in hair population density around the navel. (**a**) and (**b**): Reprinted from ref.^2^ with permission from Elsevier. (**c**) Scanning electron micrograph of a human hair fiber: Reprinted from ref.^17^ with permission from Elsevier. Note the overlapping cuticle layers. (**d**) Schematic diagram showing the dimensions of the human hair cuticle layers. Reprinted from ref.^18^ with permission from John Wiley and Sons.

Based on an online survey^1^ conducted by Dr Karl Kruszelnicki, about 83% of men and 43% of women produce belly button lint (BBL) or navel fluff; a compact tuft of lint fibers that forms in the navel. The online survey gathered further information, mostly qualitative, from the 4799 participants regarding BBL. He concluded, among other things, that having sufficient amount of body hair is an essential condition for the production of BBL (see Fig. 1a,b). Later, Steinhauser^2^ carried out chemical analysis of samples of his own BBL to reveal that its major constituent is textile fibers, confirming the general belief among the public. He also reported the presence of foreign materials such as cutaneous scales, house-dust, sweat, etc. The average mass of BBL was found to be 1.82 mg. To the best of the author’s knowledge, these are the only two formal studies on BBL.

At any rate, these articles did not attempt to perform any analysis of the physical mechanism underlying the phenomenon of BBL formation. Both the articles promoted the “Hair Transport Theory,” which suggests that the body hair (on the chest and abdomen) may act like a “one-way street” that directs the fibers that are dislodged form the clothing (via abrasion) to the navel. Steinhauser^2^ further noted that the scaly structure and the direction of growth of the hairs and normal body movement could play a major role in the dislodging and transport of lint fibers. But finer details of the mechanism and how all these factors lead to a constant driving force for the one-way transport remain elusive. Building upon these ideas, we attempt to model the phenomenon of BBL development by considering the dynamics of transport of textile fibers through the region between the piece of clothing (simply referred to hereafter as shirt, but note that it could be a piece of lower clothing as well) and skin. A periodic forcing on the fibers is proposed to be generated via the respiratory cycle. The cuticle scales on the abdominal hair act like small ratchets pointed towards the navel effects. We model this feature as an asymmetric tribological property of the hair. By solving the one-dimensional transport equation based on a uniform transport rate of the fibers, the accretion rate of lint in the navel is evaluated. We obtain a reasonable match for the mass of the BBL with the values reported experimentally by Steinhauser^2^. Other rarer body movements that could result in high amplitude forcing function (e.g. sighing) are not considered in the model. However, all these effects are expected to only enhance the transport rate of lint to the navel; hence our results are conservative. Understanding this phenomenon is of more than academic interest; the theory proposed in this paper is hoped to provide new ideas for design improvements/innovations in various technological applications, e.g., lint removal brushes, micro-tribological systems, and surfaces with anisotropic dry adhesion and wetting characteristics^3^. Also, designing more efficient lint removers are important in medical field, given that lint contamination during surgical procedures is found to cause post-operative complications^4^.

Before moving on to the theoretical framework, it is necessary to address its conceptual underpinnings. First, let us discuss the pertinent morphological features of human hair. A human hair consists of a central core (called the cortex) covered by a protective sheath of thin cellular layers (called the cuticle). As revealed by scanning electron microscope (SEM) and atomic force microscopy (AFM) images^5,6^ of a human hair fiber, the cuticle has a multi-layered structure, which is reminiscent of overlapping roof tiles (see Fig. 1c). The cuticle sheets run and overlap longitudinally in a root-to-tip direction along the hair fiber axis. Each cuticle sheet is typically 0.5 µm thick and has an exposed length of 5–10 µm (cf. Fig. 1d). Expectedly, the ratchet-like architecture of hair surface results in a strong directional dependence of the frictional properties of hair. For example, the dynamic coefficient of friction measured in the tip-to-root direction is more than twice that in the root-to-tip direction^5,6^. This is expected because the protruding edges of the cuticle scales offer additional resistance to the motion of another surface on the hair surface in the tip-to-root direction. On the other hand, the frictional resistance will be less in the root-to-tip direction. As discussed below, this directional asymmetry plays a pivotal role in facilitating the unidirectional transport of lint fibers.

The saw-tooth like topology of hair surface could also be the reason for extricating lint fibers from fabrics through abrasion, when the fabric moves relative to the hair in the tip-to-root direction. The fabric could be the shirt worn by the individual or the drying towel used after shower. Such dislodged lint fibers present over the abdominal skin finally get accumulated in the navel to form the BBL. As can be seen from the SEM image^7^, the typical width of a lint fiber constituting BBL is of the order of 10 µm. Note that the possibility of drying towel acting as a source of lint fibers is not considered by any of the previous studies, which consider only the shirt worn by the individual as the source. While this could possibly explain the rare cases of discrepancy between the color of BBL and the shirt worn, as we will see later, the contribution from lint fibers of drying towel is very small in the production of BBL.

**Figure 2:**
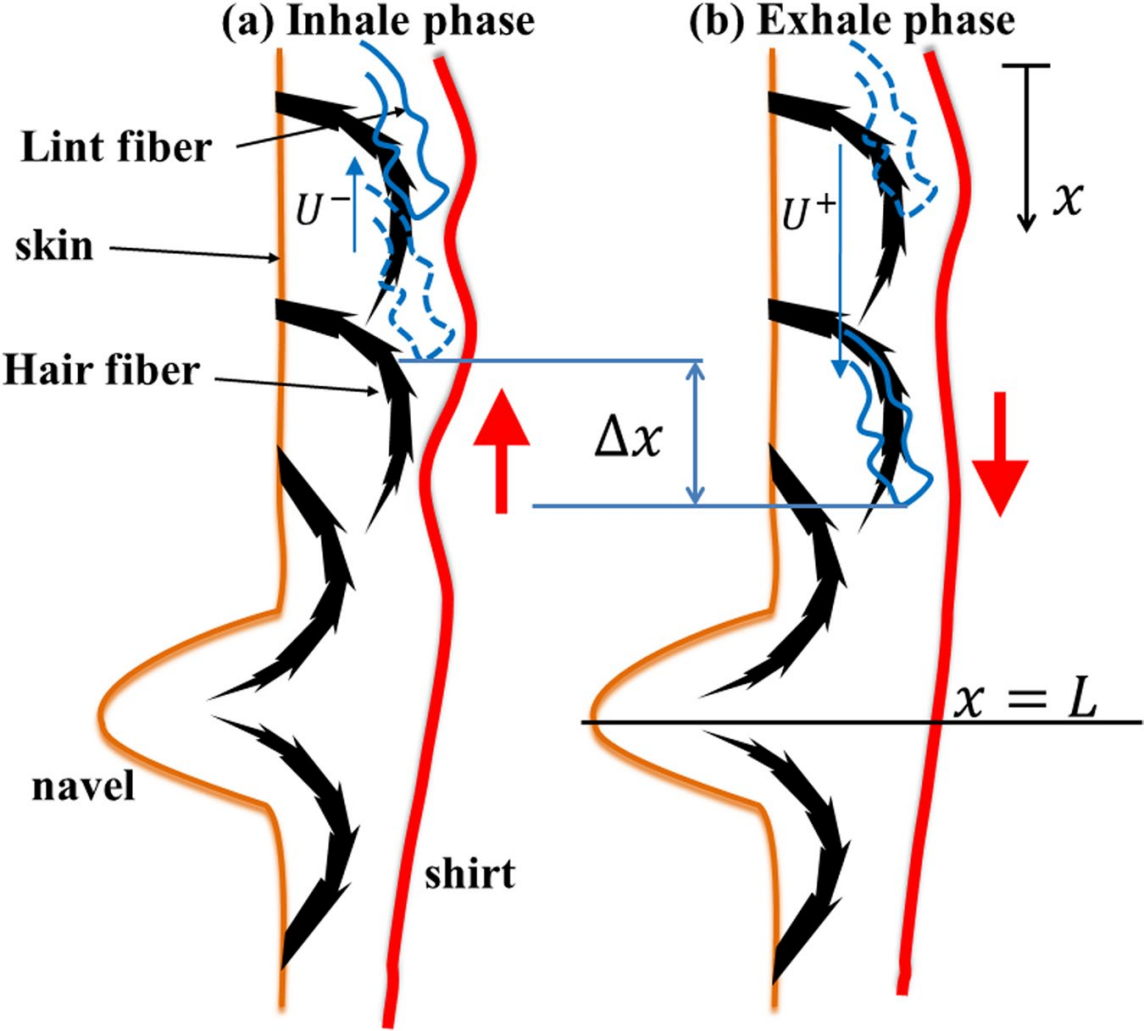
A cartoon illustrating the sliding motion of a lint fiber in an area superior to the navel. Two consecutive phases of a breathing cycle: (**a**) inhale, (**b**) exhale are shown. The spikes on the hair fibers represent the cuticle scale edges. The net effect of the relative oscillatory motion of the fabric, as shown by the red arrow, is to transport the lint fiber toward the navel in a cycle because the hairs point toward the navel. The final (initial, respectively) location of the lint in each phase is shown by the solid (dashed) curve. Since the frictional force will be more in the tip-to-root direction the sliding velocity of the lint fiber in the exhale phase (shown by the blue arrow) is less in the inhale phase than it is in the exhale phase. $\Delta x$ denotes the net displacement of the lint fiber in a cycle.

Now taking into consideration the periodic abdominal motion induced by the breathing cycle and the fact that all body hairs in the area around the navel are stooped toward the navel (see Figs 1a and 2), we arrive at a simple physical picture explaining the transport process of lint fibers to the navel. During the respiratory cycle, the size of the abdomen periodically changes, setting up a relative motion between the skin and the shirt (whether loose or tight). Let us focus on the consequences of this motion along the line connecting the navel and the middle of the chest. Similar arguments can be applied to other directions too, but are not addressed here to keep the discussion short. During the inhale phase of the respiratory cycle, in the upright position of the body, the motion of shirt is upwards relative to the skin (see Fig. 2a) and against the stooped direction of the abdominal hairs.

Consider a lint fiber (dislodged from the shirt) that is sandwiched between a hair fiber and the shirt worn by the individual. As the shirt moves relative to the hairs, the lint fiber will tend to move along with shirt. In the inhale phase, when the shirt moves against the stooped direction the hair fiber (i.e. against the overlapping cuticle scales), the lint fiber will feel more frictional resistance and consequently will have a lesser sliding velocity (Fig. 2a). Note that since the thickness of the cuticle scales is about two orders of magnitude lesser than that of the lint fiber, the chances of the lint fiber getting snagged to the cuticle scale are very small. Instead, the lint fiber will slide over the microscopic protuberances, facing higher friction. Subsequently, in the exhale phase of the respiratory cycle the shirt moves along the direction of the orientation of the hair (see Fig. 2b), the lint fiber experiences less friction in the root-to-tip direction and consequently a higher sliding velocity. As the next respiratory cycle begins, the direction of relative motion of the shirt reverses and the cycle repeats.

Since the net sliding velocity in a cycle will be directed in the root-to-tip direction, the lint fiber makes an incremental advance in the root-to-tip direction of the hairs in every breathing cycle. Note that for smooth transition of lint fiber between two cosecutive hair fibers, the hair density must be high enough to ensure that the adjacent hair fibers are overlapping (as shown in Fig. 2). This explains why bellies with sparsely populated hair (as in Fig. 1b) do not produce BBL. Thus over time, the lint fiber will be transported toward the navel, where it becomes trapped. The reason for this trapping becomes apparent in a close inspection of Fig. 1a. The hairs in the immediate vicinity of the navel stoop towards the depression of the navel, as depicted in Fig. 2. This causes the lint fiber to proceed deep enough into the depression, when its contact with the fabric will be lost and thereby the traction force on the lint fiber vanishes. This also explains why navels that stick out (colloquially called outties) rarely collect lint^1^ as in such cases the contact between the shirt and lint fibers always exist and there is no deep repository for the buildup of BBL. The continuous influx of lint fibers from all the directions around a lint-collecting navel leads to an accretion of lint fibers in the navel. Over time, the accumulated lint fibers mix with sweat and other foreign matter to form a compact mass of navel fluff.

Now let us turn to the theoretical analysis of the phenomenon. We wish to mathematically model the transport process of the lint fiber population on the body and thus provide a reasonable estimate of the accretion rate of the fibers in the navel. This will help us to predict the mass of the BBL as a function of time and thus verify our model by comparing our results with the experimentally obtained data on the mass of BBL^2^.

**Figure 3:**
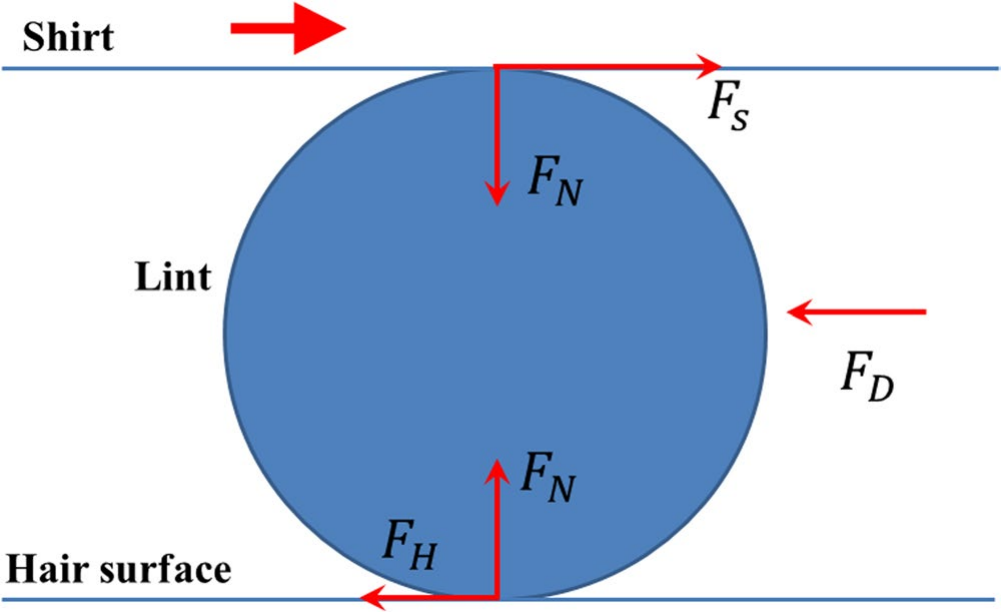
Cross-sectional view (represented by the circle) of the lint showing the different forces acting on it. Note that since the fabric is moving rightward, the frictional force exerted by the lint on the fabric will be leftward. Consequently, the frictional force exerted by the fabric on the lint will be rightward as shown by the arrow marked ${F}_{S}$. Similarly, at the hair-lint interface since the motion of the lint with respect to the hair is rightward, the frictional force ${F}_{H}$ exerted by the hair on the lint will be leftward.

We begin by examining the different forces in play in the transport of a lint fiber to understand its transport rate. As depicted in Fig. 3, since the lint fiber is sandwiched between a hair surface and a fabric surface, there will be normal forces acting at the two interfacial points in the direction normal to the direction of sliding of the lint fiber. Since we disregard any acceleration of the fiber in the normal direction, a simple force balance along this direction will show that the normal forces at both the interfaces should be the same, denoted by ${F}_{N}$. These normal forces lead to frictional forces in the sliding direction at the contact points. The frictional force at the shirt-lint contact point is denoted by ${F}_{S}$ and that at the hair-lint contact by ${F}_{H}$. Note that since the shirt is moving in the sliding direction, ${F}_{S}$ acts in the sliding direction and ${F}_{H}$ opposite to the sliding direction. These forces are given by the following expressions:

1 $${F}_{S}={\mu }_{S-L}{F}_{N}\,{\rm{and}}\,{F}_{H}={\mu }_{H-L}{F}_{N},$$

where ${\mu }_{S-L}$ denotes the coefficient of kinetic friction between the shirt and lint fiber and ${\mu }_{H-L}$ denotes that between the hair and the lint fiber. Thus the net traction force acting on the fiber is

2 $${F}_{T}={F}_{S}-{F}_{H}=({\mu }_{S-L}-{\mu }_{H-L}){F}_{N},$$

The major contribution to ${F}_{N}$ is expected to come from the pressure exerted by the clothing on the skin. Even wearing loose garments cause a skin pressure,$\,p$ of the order of 0.1 kPa, while working in the daytime^8^. This finite skin pressure induced by the clothing suggests that the fabric is under tension. To obtain a first order estimate of this tension, one may use the Laplace equation^9^

3 $$p=\gamma /R,$$

where $\gamma$ and $R$ denote the tension per unit length of the fabric and the radius of curvature of the fabric respectively. This relation can be obtained by a simple force balance and is similar to that of the pressure jump across a curved cylindrical interface between a fluid and a gas or the circumferential (hoop) stress in a thin-shelled cylinder. The analogy to the fluid system stems from the fact that due to surface tension a fluid-gas interface will also behave like an elastic membrane under tension. The tension in the fluid interface is balanced by force due to excess internal fluid pressure, while the tension in the garment is balanced by force due to skin pressure. For an arbitrary surface, the factor $1/R$ in Eq. (3) must be replaced with the sum of principal curvatures of the surface. However to obtain a conservative estimate of $\gamma$, one may consider the greater principal radius of curvature as $R$. If the shirt assumes the same surface geometry as the abdominal wall, then $R$ corresponds to the radius of curvature of the abdominal wall in the longitudinal direction, which is about 470 mm^10^ (as measured from MRI images of abdomen). Thus, we get $\gamma \approx 47$ N/m.

Assuming that the lint is also under the same amount of pressure, the normal force transmitted to the lint fiber from the shirt is given by

4 $${F}_{N}=p{A}_{c},$$

where ${A}_{c}$ is the real area of contact of the lint with the fabric. If we idealize the lint-fabric system as a cylinder in contact with a flat surface (as in Fig. 3) then the real (as well as apparent) area of contact between the surfaces is, theoretically speaking, zero. However, in practice, the roughness on the surfaces and localized solid deformation at the contact points will result in a finite real area of contact. Accurate determination of the real contact area remains a major challenge in tribological calculations^11^. Hence we approximate the real contact area to be a small fraction, $\alpha$ of the projected area of the lint fiber, $dl$, where $d$ and $l$ are the diameter (or width) and length of the lint fiber, i.e. ${A}_{c}=\,\alpha dl$. In the subsequent analysis we take $\alpha =\,{10}^{-5}$, the smallness of which is not surprising from the above arguments.

As the lint fiber moves under the action of the traction force, a relative flow of air is setup around the fiber causing the fiber to experience a drag force, ${F}_{D}$. Due to the small length scales of the fiber, we can safely assume that Stokes’ law governs this drag force; in other words, the drag force is generated by the viscous stresses exerted by the surrounding fluid on the solid surface of the fiber. Newton’s law of viscosity sets the scale for this viscous stress as $\tau  \sim \eta U/d$. Here $\eta$ is the dynamic viscosity of air and $U$ is the magnitude of the relative velocity between air and the fiber. Since this stress acts on the surface area that scales as $A \sim dl$, the drag force is given by $\,{F}_{D} \sim \eta lU$. A balance between the net traction force ${F}_{T}$ and the air drag force ${F}_{D}$ gives the scaling for the velocity of the lint fiber as

5 $${U}^{\pm } \sim ({\mu }_{S-L}-{{\mu }_{H-L}}^{\pm }){F}_{N}/\eta l.$$

Here the subscript $\pm$ is used to denote the directional friction effect of human hair^12^. The coefficient of friction between hair and lint, ${\mu }_{H-L}$ shows directional asymmetry. ${{\mu }_{H-L}}^{-}$ denotes coefficient of friction in the tip-to-root direction and can be twice that in the root-to-tip direction, ${{\mu }_{H-L}}^{+}$ ^5,6^. As explained earlier, this is attributed to the fact that the cuticle layers are aligned in the root-to-tip direction; hence the friction is weaker in that direction. Consequently, as per Eq. (5) the velocity in positive sliding direction, ${U}^{+}$ will be greater than that in the negative sliding direction ${U}^{-}$ (as shown schematically in Fig. 2). Assuming that the inhale and exhale phase are of the same duration, the average velocity of a lint fiber in a breathing cycle is then given by

6 $$\bar{u}=({U}^{+}-{U}^{-})/2 \sim ({{\mu }_{H-L}}^{-}-{{\mu }_{H-L}}^{+}){F}_{N}/2\eta l.$$

Since ${{\mu }_{H-L}}^{-}\approx 2{{\mu }_{H-L}}^{+}$, $\bar{u}\approx {{\mu }_{H-L}}^{+}{F}_{N}/2\eta l$ is positive, i.e., the net sliding velocity of the lint fiber is from the root-to-tip direction of the hair fibers. We remark that the sliding motion between two dry surfaces can exhibit very rich and complicated dynamical features such as stick-slip and chaotic behavior^12^, which are disregarded in this simple model. Further, we neglect other possible secondary events that are likely to occur during sliding such as elastic deformation of the fiber, its rotational movement, and any change in its orientation with respect to hairs.

Even though the weight of a lint fiber is comparable to the garment pressure force, we do not include gravity in the above force balance due to the following reasons. The results from the survey conducted by Dr Karl Kruszelnicki^1^ strongly suggest that the lint can migrate in a direction opposite to that of gravity and thus it plays little role in lint transport to the navel. Reports of the color of the BBL matching with that of one’s underpants and shaving off a horizontal strip of hair from one’s lower abdomen preventing the BBL production show that the trail of hair extending from the pubic region to the navel (commonly called “happy trail”) indeed facilitate a transport of lint fibers up against gravity.

To obtain a numerical estimate for the average velocity, we consider the shirt material to be cotton. Coefficient of friction between cotton and human hair, is reported to be approximately 0.3^13^, hence we take ${{\mu }_{H-L}}^{+} \sim O(0.1)$. SEM of BBL^1^ reveals that each individual lint fiber is at least 1 mm long. From a visual inspection of the individual fibers from BBL, it is apparent that they can be a few millimeters long. As we are interested only in the order of magnitude, we use $l\, \sim \,1$ mm in the analysis. This gives $\bar{u}\, \sim \,O({10}^{-5}\,m/s)$. The numerical values of the parameters used are summarized in Table 1.

**Table 1:** Notation and values of parameters used in the analysis.

Parameter	Description	Value
$A$ (m^−1^s^−1^)	Source term for BBL production	1
$d$ (m)	Width of lint fiber	10^−5^
$l$ (m)	Length of lint fiber	10^−3^
$L$ (cm)	Length of the domain considered	10
${n}_{0}$ (m^−1^)	Constant initial lint fiber distribution	100
$p$ (kPa)	Pressure due to clothing	0.1
$T$ (s)	Time period of a breathing cycle	6
$\alpha$	Ratio of real contact area to projected area of lint fiber	10^−5^
$\eta$ (Ns/m^2^)	Viscosity of air	1.84 × 10^−5^
${{\mu }_{H-L}}^{+}$	Coefficient of kinetic friction between hair and cotton in the root-to-tip direction	0.1
$\rho$ (kg/m^3^)	mass density of cotton	450

Next, let us consider the equation governing the evolution of concentration of lint fibers on the body at a given time and space. As noted earlier, for simplicity, we focus only on the motion of lint fibers along one direction (say, along the midline connecting the navel to the middle of the chest), allowing a one-dimensional approach. The generalization to two dimensions, e.g. using a polar coordinate system with the origin at the navel, is straightforward. Let $n(x,t)$ denotes the concentration (number per unit length) of lint fiber present on the bed of body hairs at the location $x$ and time $t$ and $u(x,t)$ be the corresponding velocity of the lint fibers. The coordinate system used is shown in Fig. 2b. We remark that we consider the variables $n$ and $u$ as continuous to make the analysis more transparent, but this would not change our results either quantitatively or qualitatively. A simple mass conservation of the lint fibers leads to (see Supplementary Material for derivation)

7 $$\frac{\partial n(x,t)}{\partial t}+\frac{\partial (un)}{\partial x}=S(x,t),$$

where $\,S(x,t)$ represents the rate of production of lint fibers on the hairs (e.g. the lint fibers dislodged from the shirt worn due to abrasion in breathing process or other body movements). Now, we assume that the lint fibers advance with the same velocity throughout the domain and thus replace $u$ with $\bar{u}$ the uniform average sliding velocity of lint fibers per breathing cycle (given by Eq. (6)). Further we shall assume the simplest form for the source function $S(x,t)$, namely a constant function, say, $A$. These assumptions are justified because the contact conditions between the skin and the shirt (which dictates both the sliding velocity and production of lint fibers) are not expected to change drastically over a short distance from the navel. With these assumptions, Eq. (7) takes the form

8 $$\frac{\partial n(x,t)}{\partial t}+\bar{u}\frac{\partial n}{\partial x}=A.$$

Subject to the initial condition $n(x,t=0)={n}_{0}(x)$, this linear first order partial differential equation can be solved (e.g. by a simple change of variables, $w=\bar{u}t-x\,{\rm{and}}\,z=x$^14^) to obtain

9 $$n(x,t)=At+{n}_{0}(x-\bar{u}t),$$

**Figure 4:**
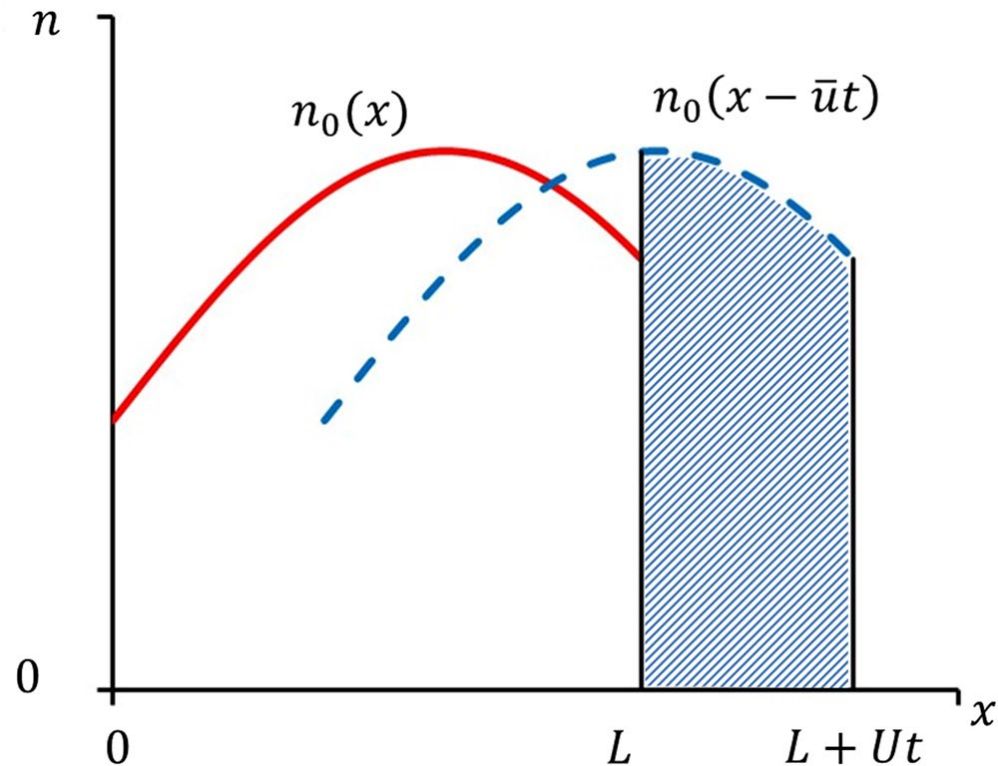
Illustration of spatiotemporal evolution of the second term on the right hand side of Eq. (9). An arbitrary initial distribution is shown by the solid (red) curve. The distribution after time, $t$ is shown by the dashed (blue) curve. For a uniform sliding speed $\bar{u}$, the distribution after time $t$ will be the initial distribution function shifted along the positive $x$ axis by $\bar{u}t$. Since $x=L$ represent the location of the navel, the number of lint fibers accrued in the navel during this time interval will be the area of the shaded portion.

The second term on the right hand side of this equation is the complimentary function of Eq. (8), i.e. the solution to first order linear wave equation. Hence it represents a translation of the initial distribution of the concentration of lint fibers, $\,{n}_{0}(x)$ along the positive $x$ direction with a speed $\bar{u}$, as time progresses (see Fig. 4). Since $t=0$ is considered as the moment when the shirt first comes in contact with the body, the major contribution to ${n}_{0}(x)$ may be considered to arise from the lint fibers generated via abrasion with the drying towel.

Given this solution, we can now calculate the influx of lint fiber to the navel. Let ${N}_{B}(t)$ denote the number of lint fibers present in the navel (located at $x=L$) at time $t$. As the lint fibers are moving at a velocity of $\bar{u}$, the number of lint fibers entering the navel over a time interval ${\rm{\Delta }}t$ will be the number of fibers within a distance $\bar{u}{\rm{\Delta }}t$ of the navel, i.e.,

10 $${N}_{B}(t+{\rm{\Delta }}t)-{N}_{B}(t)=n(x=L,t)\bar{u}{\rm{\Delta }}t,$$

Dividing by ${\rm{\Delta }}t$ and taking the limit as ${\rm{\Delta }}t$ approaches $0$ gives the following rate equation for the number of lint fibers present in the navel

11 $${\dot{N}}_{B}(t)=n(x=L,t)\bar{u},$$

with the over-dot indicating differentiation with respect to $t$. Hence, as initially (at $t=0$) the navel is devoid of any lint fibers, in time $t$ the number of lint fibers accumulated in the navel is given by

12 $${N}_{B}(t)=\bar{u}{\int }_{0}^{t}n(x=L,\hat{t})d\hat{t},$$

where $\hat{t}$ is a dummy variable of integration. Using Eq. (9) we get

13 $${N}_{B}(t)=\{\begin{array}{c}\frac{A\bar{u}{t}^{2}}{2}+{\int }_{L-\bar{u}t}^{L}{n}_{0}(x)dx\,{\rm{f}}{\rm{o}}{\rm{r}}\,t < \frac{L}{\bar{u}}\\ \frac{A\bar{u}{t}^{2}}{2}+{\int }_{0}^{L}{n}_{0}(x)dx\,{\rm{f}}{\rm{o}}{\rm{r}}\,t\ge \frac{L}{\bar{u}}\end{array}.$$

Finally, the mass of BBL as a function of time is given by

14 $${m}_{B}(t)={m}_{L}{N}_{B}(t),$$

where ${m}_{L}\approx \rho \pi {d}^{2}l/4$ is the mass of one lint fiber.

Note that the second term on the right hand side of Eq. (13) becomes a constant (i.e. independent of $t$) for times $t > \frac{L}{\bar{u}}$. The reason is that this part of the solution represents, as discussed earlier, the contribution to the BBL from the propagation of lint fiber concentration, $\,{n}_{0}(x)$ initially present on the domain of length $L$. With reference to Fig. 4, it becomes evident that in time $t=\frac{L}{\bar{u}}$, all the initially present lint fibers, which amounts to ${\int }_{0}^{L}{n}_{0}(x)dx$, will reach the navel.

We consider the asymptotic behaviour of the solution given by Eqs (13) and (14) at very small times and at large times. Further, we can assume that the estimate of the initial distribution is reasonably close to a constant, i.e.$\,\,{n}_{0}(x)=\,{n}_{0}$ in the interval $0 < x < L$, because the hair population density is almost uniform near the navel and hence the probability of lint fibers getting snagged during abrasion with a drying towel will be almost uniform. Thus, we have

15 $${N}_{B}(t)=\{\begin{array}{c}\frac{A\bar{u}{t}^{2}}{2}+\bar{u}{n}_{0}t\,{\rm{f}}{\rm{o}}{\rm{r}}\,t < \frac{L}{\bar{u}}\\ \frac{A\bar{u}{t}^{2}}{2}+{n}_{0}L\,{\rm{f}}{\rm{o}}{\rm{r}}\,t > \frac{L}{\bar{u}}\end{array},$$

Clearly, in the long-time limit (for arbitrary large $t$), the parabolic term on the right hand side of Eq. (15) will dominate over the constant second term. Consequently, we get

16 $${m}_{B}(t\to \infty )=\frac{{m}_{L}A\bar{u}{t}^{2}}{2}.$$

Since ${n}_{0}$ does not appear in this expression, it is clear that the contribution of the initially present lint fibers to BBL production becomes vanishingly small at large times. This is corroborated by the previously made observations^1,2^ that the color of BBL matches the shirt that is worn. In other words, the lint fibers constituting the BBL mostly originate from the shirt worn, or, the source term is the major contributing factor for BBL production. The experimental data available from Steinhauser^2^ is from the specimens that he collected *at the end of the day*. Hence, if we consider that the specimens were produced over a time span of, say, 10 hours, then $t\, \sim \,{10}^{4}\,\,$s. At such large times, we expect that Eq. (16) will predict the evolution of BBL.

Note that $A$ represents the rate of formation of lint fibers per unit length of the domain and expectedly this will be a strong function of the grament pressure, or equivalently, the skin pressure. For example, a tighter shirt would imply higher probability of lint fibers getting dislodged from the cloth. However, due to lack of further information, we evaluate $A$ based on the following heuristic arguments. Suppose that during each breathing cycle (of period $T\approx 6\,s$), a lint fiber is added to the domain of length $L=10$ cm; this gives $A \sim 1$ m^−1^s^−1^. Subsequently, ${m}_{B}(t=10\,hrs)\, \sim \,1$ mg, whose order of magnitude is in agreement with the experimentally reported value of 1.8 mg. Note that we did not consider the presence of foreign matter observed in BBL’s such as fat, proteins, cutaneous scales, etc. Also the one-dimensional model considers the lint fibers coming into the navel from just one direction. Effects of these factors on the above result can be absorbed in the uncertainty of our knowledge of $A$.

For completeness, we now look at the short-time limit, where the second (linear) term is important; thus

17 $${m}_{B}(t\to 0)={m}_{L}\bar{u}{n}_{0}t.$$

Since this part of the solution has little contribution to the mass of BBL at large times, only a crude estimate of ${n}_{0}$ is sufficient to evaluate this expression. We presume that ${n}_{0}$ is of the same order as the population density of body hair, hence ${n}_{0}\approx 100$ m^−1^; this is equivalent to assuming that initially one lint fiber is attached to each hair fiber. Then Eq. (17) predicts a very slow linear growth rate of BBL; even at time $t \sim \frac{L}{\bar{u}}=1$ hr, this predicts a mass of BBL of the order of 0.1 µg. Hence it is clear that for this part of the solution to be comparable with the contribution from the parabolic term at large times, ${n}_{0}$ has to be unrealistically large, thus corroborating our previous argument.

To further corroborate the validity of our model, we now provide more qualitative arguments showing how the model is consistent with a variety of experimental observations previously made^1,2^. For instance, it is reported that aged and more physically active individuals show more propensity to produce BBL. Since, as per our model, breathing is the driving force for the production of BBL, the increase in respiratory rate with age^15^ and physical activity explains this observation. The model also reveals the reasons for the observation that larger bellies accumulate more BBL. In addition to the obvious reason of larger collecting surface (which enhances the magnitude of the term ${n}_{0}L$ in Eq. (15)), a larger belly has lower radius of curvature and thus for the same fabric tension this leads to a higher skin pressure according to Eq. (3). This in turn result in an increase in the traction force experienced by lint fibers and a subsequent enhancement in their transport rate, $\bar{u}$. Further, the probability of lint fibers being dislodged from the shirt also increases, thus enhancing the production term, $A$ in Eq. (7). In passing, we comment that breathing-induced transport of lint fibers must be occurring in other hairy regions of the upper body that is in contact with fabric. For instance, the observation of tint fluff similar to BBL in the intergluteal cleft^1^ is also consistent with the physical picture presented in this study, given that the body hairs in regions superior to the intergluteal cleft is oriented toward the cleft.

Our results help to disprove the conjecture that the build-up of static-charge could play a role in the BBL production. It is possible that the lint fiber will accumulate static charge due to friction. However, static-charge build-up cannot lead to a unidirectional transport of lint fibers to the navel, unless there exists a well-coordinated distribution of charge on the skin/shirt that provides a constant driving force toward the navel; this sounds implausible. Further, since all the lint fibers are expected to have the same kind of charge, this theory also cannot explain the eventual amalgamation of all the fibers in one location. Similarly, given the microscopic dimensions of lint fiber, one could reason that intermolecular surface forces (e.g. van der Waals forces) are important in this phenomenon. In order to obtain a feel for the magnitude of these forces, we compared the experimentally measured pressure due to adhesive forces between lubricated ultra-smooth silicon surfaces^16^ with the skin pressure induced by garment; both are found to be of the same order. However, since the surfaces of lint fiber, cloth and hair are not smooth and the area of surfaces in contact is very little, the magnitude of short-range forces is expected to be negligible in the present problem. Notwithstanding, the effect of such forces can be accommodated in the present model by suitably changing the pressure experienced by the lint fiber (in Eq. (4)).

To conclude, we have presented a theoretical model to understand the phenomenon of formation of navel fluff. We hypothesize that the combined action of the oscillatory motion of the shirt induced by breathing and the microscopical saw-tooth serrations (due to the overlapping cuticle scales) on the abdominal hairs stooping towards the navel leads to a perpetual production of lint fibers on the body, which is channeled to the navel, where the BBL builds up. Our model quantifies this hypothesis and shows that the mass of BBL in the navel increases linearly with time initially (Eq. (17)) and at large times it grows quadratically with time (Eq. (16)) for a constant uniform source term. The value of the source term, $A$ is obtained by comparing the theoretical results with experimentally reported mass of BBL. The effect of lint fibers present initially on the body (e.g. formed via abrasion with drying towel after shower) is found to have little contribution to BBL production. Further, the model captures all the essential features of the physics underlying the mechanism producing navel fluff and is consistent with all the observations reported in the literature regarding BBL. This simplified model offers a theoretical framework that provides a conclusive explanation of the phenomenon, both qualitatively and quantitatively. We hope that this preliminary model will stimulate experimental studies that will provide detailed descriptions of the source terms and initial conditions, thus supplementing the model.

## Electronic supplementary material

Supplementary Information
