# Supplementary material for: Modeling the production of belly button lint
Source: Sci Rep. 2018 Sep 27;8:14472. doi: 10.1038/s41598-018-32765-9 (PMC6160492; doi:10.1038/s41598-018-32765-9)
Supplement: Supplementary file 1 — Supplementary Information [file 41598_2018_32765_MOESM1_ESM.pdf]

Supplementary Information for

Modeling the production of belly button lint

P. Deepu
Department of Mechanical Engineering, Indian Institute of Technology Patna, Bihta, 801103, Bihar, India

Correspondence should be addressed to deepu@iitp.ac.in

Derivation of Eq. (7)

Consider a one-dimensional element of thickness Δx centered at the point x bounded by the points $x - \frac{\Delta x}{2}$ and $x + \frac{\Delta x}{2}$. Over a time interval Δt centered around t, the mass conservation of lint fibers in this element can be stated as

$$\text{mass stored} = \text{mass in} - \text{mas out} + \text{mass generated}. \qquad \text{(S1)}$$

Each term in this equation is given as follows.

$$\text{mass stored} = m_L \left[n\left(x, t + \frac{\Delta t}{2}\right) - n\left(x, t - \frac{\Delta t}{2}\right) \right] \Delta x, \qquad \text{(S2)}$$

where m_L is the mass per lint fiber. Assuming velocity u to be positive (i.e. directed toward positive x direction),

$$\text{mass in} = m_L n\left(x - \frac{\Delta x}{2}, t\right) u\left(x - \frac{\Delta x}{2}, t\right) \Delta t, \qquad \text{(S3)}$$

because in time Δt the mass within the volume of $u\left(x - \frac{\Delta x}{2}, t\right) \Delta t$ will enter the element through the location $x - \frac{\Delta x}{2}$. Similarly

$$\text{mass out} = m_L n\left(x + \frac{\Delta x}{2}, t\right) u\left(x + \frac{\Delta x}{2}, t\right) \Delta t. \qquad \text{(S4)}$$

Finally,

$$\text{mass generated} = m_L S(x, t) \Delta x \Delta t. \qquad \text{(S5)}$$

Hence Eq. (S1) read

$$m_L \left[n\left(x, t + \frac{\Delta t}{2}\right) - n\left(x, t - \frac{\Delta t}{2}\right) \right] \Delta x = \left\{ \begin{matrix} m_L n\left(x - \frac{\Delta x}{2}, t\right) u\left(x - \frac{\Delta x}{2}, t\right) \Delta t - m_L n\left(x + \frac{\Delta x}{2}, t\right) u\left(x + \frac{\Delta x}{2}, t\right) \Delta t \\ + m_L S(x, t) \Delta x \Delta t \end{matrix} \right., \qquad \text{(S6)}$$

Dividing this equation by $m_L \Delta x \Delta t$ and taking the limit as both Δx and Δt approach 0, one obtains Eq. (7).
